# Leadership, burnout, and organizational mental health: a neuroscience-informed perspective

**DOI:** 10.3389/fpubh.2026.1881851

**Published:** 2026-10-09

**Authors:** Gisele Kitayama, Federico Gerardo de Cosio

**Affiliations:** 1 PAHO Country Office, Pan American Health Organization, La Paz, Bolivia; 2 Retired, Pan American Health Organization/World Health Organization, Washington, DC, United States

**Keywords:** leadership, mental health, neurosciences, occupational burnout, occupational health

## Abstract

This article argues that the persistent burden of burnout reflects a fundamental conceptual problem—burnout continues to be framed primarily as an individual condition rather than as an occupational phenomenon shaped by interacting structural, organizational, and leadership conditions. Burnout has emerged as a major occupational health challenge. In a 2024 survey across eight predominantly high and upper-middle-income countries, 48% of respondents reported currently grappling with burnout. Separately, depression and anxiety cost the global economy approximately one trillion dollars annually in lost productivity. Drawing on evidence from neuroimaging, stress biology, cognitive neuroscience, and organizational research, this article examines plausible pathways through which leadership behavior may influence conditions that contribute to or protect against burnout, including social threat–reward processing, cognitive flexibility, emotional intelligence, psychological safety, emotional contagion, and stress-related neurobiological responses. Among physicians, burnout has been associated with depression and anxiety, while evidence regarding substance abuse is less consistent. These proposed mechanisms are proximal and potentially modifiable, but operate within broader structural conditions that constrain or enable their effects. Human-centered work design and neuroleadership-informed leadership practices are proposed as complements to broader structural interventions, with implications for health systems, workforce sustainability, and policy design.

## 1 Rethinking burnout: from an individual phenomenon to an organizational and leadership responsibility

Neuroscience and leadership research have converged over the past two decades into the field of neuroleadership, which has concentrated principally on leadership development, decision-making, and organizational performance (1, 2). Neuroimaging studies suggest that leadership experiences can be associated with distinct patterns of brain activity (1, 3). Reviews of the field nonetheless observe that attempts to translate neuroscientific findings into leadership practice have been only moderately successful, and that several areas remain understudied (1, 2). Comparatively little of this work has addressed organizational mental health, and burnout in particular, as an outcome. This article addresses that gap by proposing an integrative conceptual framework that examines how leadership behavior may influence organizational mental health through plausible neurobiological pathways operating within broader structural conditions.

Advances in neuroscience have deepened understanding of how individuals perceive, interpret, and respond to workplace environments—and how leaders shape those environments through their behavior and decisions (1). These advances have opened a new interdisciplinary field called neuroleadership, which translates neuroscientific findings into practical leadership tools (4, 5).

In recent years, mental health has gained unprecedented visibility, particularly in the wake of the COVID-19 pandemic. However, this awareness has not translated into meaningful reduction of the problem—and burnout sits at the heart of this gap. Burnout, emotional exhaustion, cynicism, and reduced professional efficacy are not peripheral concerns: they are expressions of a broader mental health crisis that remains systemically neglected. Median government spending on mental health remains at just 2% of total health budgets (6), unchanged since 2017. Of that already limited allocation, roughly two-thirds goes to stand-alone psychiatric hospitals with services delivered mostly in institutional rather than to the community-based services where people are best served (6).

Neuroscience and neuroleadership have become particularly relevant here: understanding the brain mechanisms behind stress, threat perception, and emotional regulation offers leaders not just awareness of burnout, but additional insights to intervene before it takes hold—shifting the response from reactive to preventive (4).

## 2 Conceptual framework: leadership and mental health from a neuroscience perspective

In this Perspective, we use organizational mental health as an integrative concept referring to the collective organizational conditions through which structures, leadership practices, interpersonal relationships, and work processes influence the psychological functioning and mental health of the workforce over time. The term is used at the organizational level rather than as a clinical diagnosis or as a substitute for established constructs. It differs from occupational mental health, which concerns workers’ mental health in relation to occupational exposures and working conditions (7), and from workplace well-being, a broader construct encompassing positive functioning, satisfaction, engagement, and quality of working life. Psychological safety is narrower still, describing the shared belief that interpersonal risk-taking is safe within a team (8)—one of several organizational conditions contributing to organizational mental health rather than its equivalent.

## 3 Literature selection

This Perspective is a narrative and conceptual synthesis across neuroscience, occupational health, organizational behavior, leadership, and public health; it was not designed as a systematic or scoping review. Literature was identified around the domains informing the proposed framework: burnout and occupational mental health; chronic stress and allostatic load; leadership and neuroleadership; social threat and reward processing; cognitive flexibility; emotional intelligence; psychological safety; emotional contagion; and human-centered work design. Priority was given to peer-reviewed systematic reviews, meta-analyses, neuroscience studies, and authoritative public-health guidance, with recent literature preferred for evolving or contested areas; selected institutional and practitioner sources were retained where they constituted the primary source for frameworks discussed or provided relevant contemporary updates, and are identified as such.

## 4 The emergence of neuroleadership

The link between leadership and neuroscience gained momentum in the early 2000s, as advances in neuroimaging and cognitive science revealed how the brain processes social interactions, decision-making, and emotional regulation (1). Key concepts emerging during this period—neuroplasticity, mirror neurons, and the brain’s threat–reward circuitry—reshaped understanding of human behavior at work (9).

Mirror neuron research suggests a neurobiological mechanism that may contribute to empathy, imitation, and emotional attunement. Early claims linking these cells directly to complex social cognition were subsequently challenged, and the field entered a period of marked skepticism (9). More recent appraisals indicate that this correction may have overshot: mirror neuron processes appear to make a demonstrable contribution to lower-level social functions such as movement copying and action discrimination, even if they do not by themselves explain higher-order phenomena such as intention reading. On this basis, current reviews call for renewed and more systematic investigation rather than abandonment of the construct (9). Bibliometric analysis of the field from 1996 to 2024 confirms that research activity has continued to evolve across multiple disciplines, with sustained attention to the relationship between mirror neuron functioning and social affective processes including empathy and social learning (10). Converging neuroimaging evidence continues to support shared neural activation during the observation and experience of social and emotional events, although the mechanisms underlying this activation extend beyond mirror neurons alone and remain incompletely resolved (9, 10). These findings carry implications for leadership. Independently of the specific neural substrate, a growing body of evidence indicates that leaders’ emotional states can influence corresponding emotional and behavioral responses in team members through processes collectively described as emotional contagion. Leaders experiencing chronic, unmanaged stress may therefore contribute to team-level emotional contagion, a plausible but not directly established pathway to collective vulnerability to burnout (11).

### 4.1 The brain’s threat–reward system

At the core of these advances is a deeper understanding of how the brain continuously evaluates its environment, including signals of safety or threat, and adjusts emotional and cognitive responses accordingly (12).

When the environment is perceived as threatening—due to ambiguity, sustained pressure, lack of recognition, or social exclusion—stress-related neural and physiological systems can trigger responses including cortisol and adrenaline, potentially affecting emotional regulation and executive functions such as decision-making, creativity, and problem-solving (12, 13). In contrast, environments that promote psychological safety, clarity, and recognition may support cognitive engagement (8, 12).

### 4.2 The SCARF model: social threat and reward

Leadership plays an important role in how the environment is perceived by those being led. The SCARF model—Status, Certainty, Autonomy, Relatedness, and Fairness—is an evidence-informed practitioner framework rather than an experimentally derived model, which proposes that these five social dimensions can engage threat–reward mechanisms also involved in responses to physical threats (14). Everyday leadership interactions—feedback, communication style, decision transparency, and recognition—can therefore influence perceived social threat and reward, with cognitive and emotional consequences for individuals and teams (14). An institutional analysis of more than 15,000 SCARF assessment responses collected in 2024–2025 reported Fairness and Autonomy as the most prominent dimensions and identified differences by organizational role, including potential blind spots among senior leaders (15). These descriptive findings update the model’s application but do not constitute independent validation of its proposed neurobiological mechanisms.

### 4.3 Cognitive flexibility

Neuroleadership encompasses two foundational capacities. The first is cognitive flexibility—the ability to shift thinking strategies and adapt to new situations. The brain supports this capacity through distributed networks including frontoparietal and cingulo-insular systems involved in detecting and implementing shifts in behavior (16). Cognitive flexibility is not simply a fixed trait, although evidence regarding its enhancement through training and transfer to real-world functioning remains mixed (16).

### 4.4 Emotional intelligence

The second foundational capacity is emotional intelligence—encompassing empathy, self-awareness, and social skills relevant to how leaders perceive, regulate, and respond to social and emotional information (17, 18). Contemporary neuroscience indicates that emotional intelligence involves distributed neural systems supporting cognitive, emotional, and social processing, although its precise neural organization remains incompletely understood (17).

### 4.5 Psychological safety as an organizational resource

Through neuroleadership tools, leaders develop awareness of how their own stress responses affect their interactions—and how, when unmanaged, these states may contribute to emotional contagion within their teams, potentially increasing collective vulnerability to burnout (11, 18). The goal of neuroleadership is therefore to equip leaders to create psychologically safe environments—defined as “the shared belief that one will not be punished or humiliated for speaking up with ideas, questions, concerns, or mistakes” (8)—where leaders and teams feel trusted, valued, and free to contribute without fear. This reduction in perceived interpersonal threat may support motivation and collaboration and may reduce sustained stress activation over time, a plausible pathway relevant to burnout (4, 8).

Leadership may therefore influence the conditions under which stress-related neurobiological responses occur; through the cumulative effect of daily interactions, these conditions can contribute to stress activation or support balance, resilience, and sustained performance (4, 12), (Figure 1).

**Figure 1:**
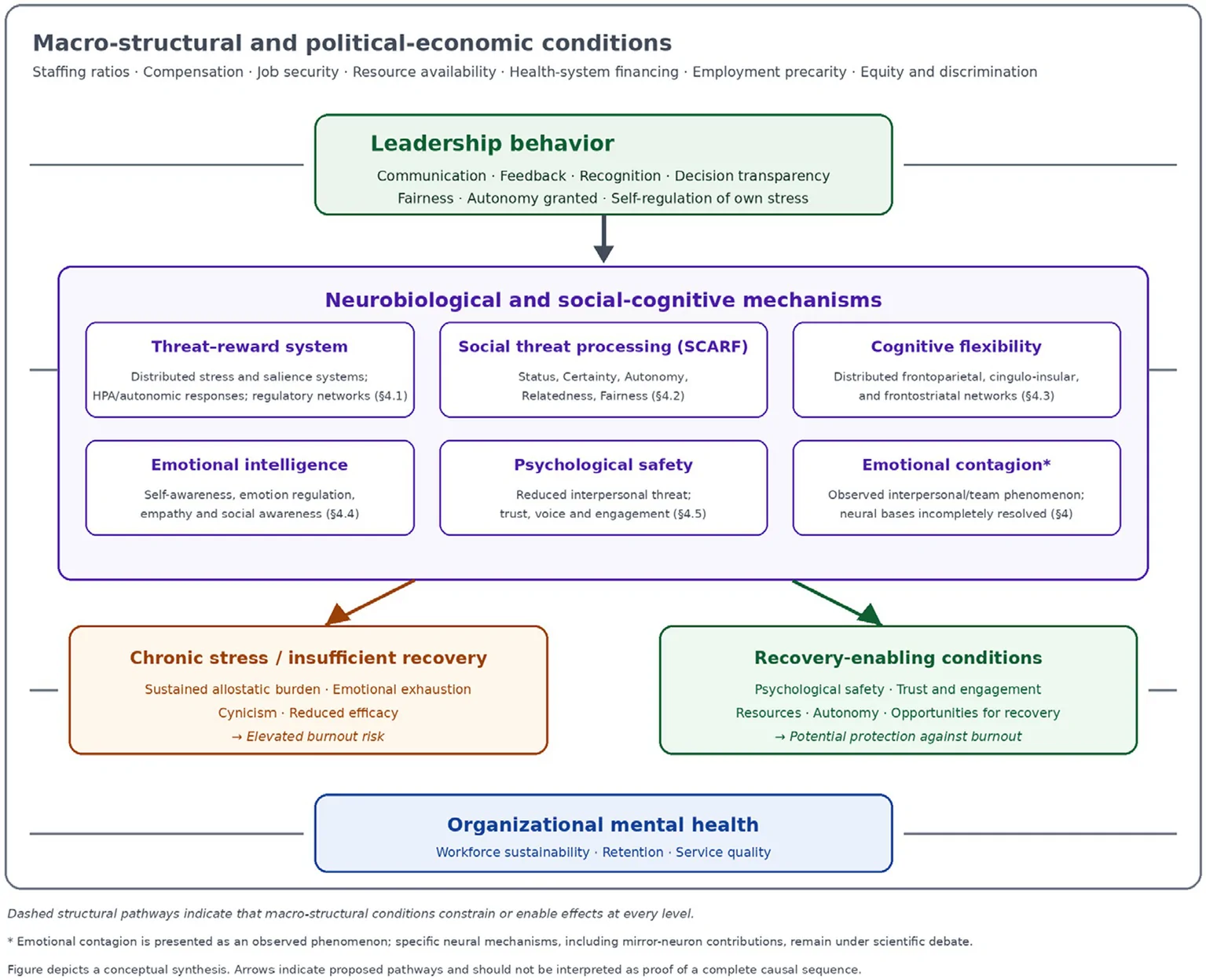
Conceptual framework linking leadership behavior to organizational mental health through neurobiological and social-cognitive mechanisms, situated within macro-structural conditions. Section numbers refer to where each mechanism is discussed in the text.

## 5 Burnout: from awareness to understanding

### 5.1 Definition and recognition

In 2019, the World Health Organization formally recognized burnout as an occupational syndrome—not a medical condition—resulting from chronic workplace stress that has not been successfully managed, characterized by three dimensions: “feelings of energy depletion or exhaustion; increased mental distance from one’s job, or feelings of cynicism; and reduced professional efficacy” (19).

This understanding did not emerge overnight. As early as 1981, Veninga and Spradley proposed a five-stage progression of burnout—from initial enthusiasm, through growing fatigue and chronic stress, into cynicism and full emotional collapse—noting that by the cynicism stage, individuals become so detached that they stop registering warning signs, including those of their own physical deterioration (20). Contemporary stress biology indicates that subjective detachment does not necessarily eliminate biological burden—allostatic load (the cumulative physiological cost of chronic stress-system activation) may continue to accumulate, with potential effects on health and cognitive functioning (12, 13).

### 5.2 Burnout as a systemic—not individual—condition

The WHO definition, while advancing recognition, also presents a persistent risk: its interpretation as solely an individual condition. Evidence indicates that structural and relational factors can contribute to burnout risk. Work environments characterized by unsustainable workloads, dysfunctional organizational cultures, and low levels of leadership support are among the conditions associated with poorer mental health and burnout-related outcomes (7, 21).

From a neurobiological perspective, burnout may develop in the context of prolonged exposure to unresolved stressors, where stress systems remain activated without adequate recovery (12, 13). Sustained stress-system activation may impair cognitive and emotional functioning over time (12, 13).

### 5.3 The progression toward crisis

When left unaddressed, burnout does not remain contained. Research among physicians indicates that burnout is associated with depression and anxiety, and most studies examining suicidality also report an association; evidence regarding substance abuse is less consistent (22, 23).

The available evidence does not establish that burnout directly causes suicide. Rather, when its underlying causes go unaddressed and associated distress continues to worsen, it can—in some individuals and circumstances—become part of a deteriorating trajectory that, in its most severe expression, may reach a point of crisis. This underscores the urgency of early organizational intervention, well before clinical thresholds are crossed (22).

## 6 Leadership as a systemic responsibility

### 6.1 The scale of the problem

Burnout remains at elevated and concerning levels across many world regions, though available data are concentrated in high-income and corporate contexts. A 2024 study by Boston Consulting Group, surveying workers across eight predominantly high and upper-middle-income countries, found that 48% of respondents reported currently grappling with burnout (24). Signs of burnout among leaders have increased 60% since 2020, with 72% of leaders reporting they often feel used up at the end of the day, and only 15% feeling prepared to prevent employee burnout in their teams (25).

Regional evidence is more limited and heterogeneous. A 2025 systematic scoping review of occupational stress syndromes among health care workers in Latin America (2019–2023) found burnout prevalence ranging from 13.2 to 70.3% across studies, reflecting wide variation in instruments and settings; within that range, a multicountry study of 5,437 health professionals from six countries reported 59.8% screening positive, with higher risk among younger professionals, women, physicians, and public-sector workers (26, 27). Together, these studies point to a substantial regional burden while also illustrating the heterogeneity of available estimates and the need to consider organizational as well as individual responses.

The economic consequences are substantial: the World Health Organization estimates that 12 billion working days are lost annually to depression and anxiety—costing the global economy approximately one trillion dollars in lost productivity (28).

### 6.2 From symptoms to causes: leadership within the system

A fundamental shift is needed—from the individual alone to the system, and from symptoms to underlying causes (7, 28). Leadership emerges as an important organizational factor—not merely a manager of processes but a shaper of environments that can amplify strain or enable clarity, trust, and meaning (7, 8).

Addressing burnout cannot be limited to isolated well-being interventions or individual coping strategies (7, 28). In neuroleadership terms, this means developing five interconnected capacities: reading the emotional and cognitive state of teams to detect early signs of strain (18); reducing unnecessary uncertainty and creating clarity, addressing predictability as an important social threat dimension (14, 15); fostering trust and psychological safety, reducing perceived interpersonal threat and supporting cooperation, since teams that feel safe raise problems early, while teams that feel threatened conceal them (8); promoting autonomy and recognition, supporting motivation (14); and regulating one’s own emotional responses, as leaders who model emotional regulation may support co-regulation in their teams through processes of emotional contagion (11, 18).

It is important to acknowledge, however, that the neurobiological mechanisms discussed in this article operate within—and are significantly shaped by—broader structural and political-economic determinants of burnout. For the purposes of this argument, three levels can be distinguished. (1) Macro-structural and political-economic: staffing ratios, compensation adequacy, job security, discriminatory workplace practices, resource scarcity, and health-system financing. (2) Organizational and leadership practice. (3) Individual neurobiological response. This article addresses primarily the second and third, while recognizing that the first is typically determined beyond the reach of individual organizations and constrains what leadership practice can achieve. Neuroleadership does not replace the need for structural reform at the macro level; rather, it offers a complementary, mechanistically informed lens through which leadership behavior can be understood as one proximal and potentially modifiable influence on individual stress-related responses—one that operates within and can either mitigate or amplify the effects of structural conditions. Recognizing this boundary is both an intellectual obligation and a practical necessity, particularly in low and middle-income country contexts where structural constraints on well-being may be most severe (7, 26, 28).

In this sense, neuroleadership is relevant not only to organizational development but also to the broader public health challenge of creating healthier work environments.

## 7 Bridging theory and practice: toward human-centered processes

Translating these insights into organizational process design is the next imperative, yet a persistent gap remains between awareness and effective transformation of work environments (7).

Part of this gap stems from an approach that has traditionally prioritized process optimization over human experience. From a neuroscience perspective, however, processes are not neutral—they are experienced and interpreted by individuals whose brains continuously evaluate environmental demands, uncertainty, safety, and threat (12, 13). The inflection point lies not in abandoning processes, but in redesigning them through a human-centered lens—and this is where neuroleadership may provide useful tools (4, 14). A human-centered approach integrates the subjective experience of work, emotional and uncertainty management, professional identity, and the quality of daily interactions (29).

The structural conditions most commonly identified as targets for systemic intervention—grounded in the WHO Guidelines on Mental Health at Work—include flexible working arrangements; workload management and reduction of excessive demands; worker participation and meaningful autonomy in decision-making; clear role definition and transparent communication from leadership; formal training of managers in mental health awareness; anti-harassment and anti-bullying policies; equitable recognition and reward systems; and structured return-to-work programs for those who have experienced mental health conditions (7). These are not merely administrative measures: several align with social threat dimensions identified in the SCARF model (14, 15). When consistently applied, they may reduce sustained stress burden and create the conditions for leaders and teams to perform sustainably and recover effectively (8, 12, 13). Comparative work on human resource management practices across regions similarly indicates that organizational structures and managerial practices shape employee well-being outcomes, while underscoring that the relative weight of these factors varies substantially by regional and institutional context (30).

Leaders themselves are equally entitled to a process of mindset change. Neuroscience indicates that the adult brain retains substantial structural and functional plasticity in response to experience (12). Through structured reflection, habit activation, and evidence-based development programs, leaders may revise aspects of their leadership orientation—one aligned with the conditions required for a healthy workplace (4, 15). Leaders who themselves experience psychological safety, clarity of purpose, and autonomy may be better positioned to extend these conditions to their teams—potentially interrupting processes through which stress can propagate from leadership to workforce (8, 11).

## 8 Limitations

This article has several important limitations that should be acknowledged. First, as a perspective piece, it synthesizes existing evidence rather than reporting original empirical findings; no study has directly tested the full proposed pathway from leadership behavior to neurobiological state to burnout outcomes in a controlled setting (1, 5). Second, the neuroleadership framework draws substantially on findings from cognitive and social neuroscience generated in laboratory and high-income country contexts—their transferability to diverse occupational settings, particularly in low and middle-income countries with severe resource constraints, systemic understaffing, or precarious employment, remains to be empirically established (26). Third, while neurobiological mechanisms are presented as proximate pathways, this article does not claim they are sufficient explanations of burnout in the absence of structural intervention (7). Fourth, several practitioner frameworks cited, including the SCARF model, have not undergone the same independent replication as the peer-reviewed neuroscience on which they draw (9, 14). The integration of leadership, neurobiological mechanisms, and burnout presented here should therefore be interpreted as a conceptual synthesis of distinct evidence streams rather than as an empirically established causal pathway. Empirical research is needed to test the neuroleadership–burnout pathway across diverse occupational and geographic contexts.

## 9 Conclusion

Advancing toward healthier work environments requires a fundamental shift in how organizations are understood and designed. Workplaces are not neutral systems of production; they are environments continuously interpreted by workers, with conditions that can influence cognitive, emotional, physiological, and mental-health outcomes (7, 12, 28). When adverse conditions are sustained, their effects can extend beyond immediate work performance and contribute to longer-term physical and mental health burden (12, 28).

The argument advanced here is that burnout is not an inevitable consequence of demanding work, but a potential outcome of environments characterized by sustained stress, inadequate recovery, and low psychological safety (8, 12, 13, 19). Conversely, supportive leadership practices and organizational structures may help mitigate some of these risk pathways, while their effects remain constrained or enabled by broader structural conditions (7, 8, 28).

The challenge ahead is not the absence of knowledge, but its translation into consistent practice. In this sense, leadership is one modifiable influence on organizational mental health and a strategic lever for shaping healthier, more resilient systems (1, 7, 8). The future challenge is no longer recognizing burnout, but redesigning organizations around the realities of human functioning.

## Data Availability

The original contributions presented in the study are included in the article/supplementary material, further inquiries can be directed to the corresponding author.
